# Single nucleotide polymorphisms affect miRNA target prediction in bovine

**DOI:** 10.1371/journal.pone.0249406

**Published:** 2021-04-21

**Authors:** Marco Antônio Perpétuo de Sousa, Flavia Regina Florêncio de Athayde, Mariângela Bueno Cordeiro Maldonado, Andressa Oliveira de Lima, Marina Rufino S. Fortes, Flavia Lombardi Lopes

**Affiliations:** 1 Department of Production and Animal Health, São Paulo State University (Unesp), School of Veterinary Medicine, Araçatuba, São Paulo, Brazil; 2 School of Chemistry and Molecular Bioscience, The University of Queensland, Brisbane, Queensland, Australia; University of Tennessee Health Science Center, UNITED STATES

## Abstract

Single nucleotide polymorphisms (SNPs) can have significant effects on phenotypic characteristics in cattle. MicroRNAs (miRNAs) are small, non-coding RNAs that act as post-transcriptional regulators by binding them to target mRNAs. In the present study, we scanned ~56 million SNPs against 1,064 bovine miRNA sequences and analyzed, *in silico*, their possible effects on target binding prediction, primary miRNA formation, association with QTL regions and the evolutionary conservation for each SNP locus. Following target prediction, we show that 71.6% of miRNA predicted targets were altered as a consequence of SNPs located within the seed region of the mature miRNAs. Next, we identified variations in the Minimum Free Energy (MFE), which represents the capacity to alter molecule stability and, consequently, miRNA maturation. A total of 48.6% of the sequences analyzed showed values within those previously reported as sufficient to alter miRNA maturation. We have also found 131 SNPs in 46 miRNAs, with altered target prediction, occurring in QTL regions. Lastly, analysis of evolutionary conservation scores for each SNP locus suggested that they have a conserved biological function through the evolutionary process. Our results suggest that SNPs in microRNAs have the potential to affect bovine phenotypes and could be of great value for genetic improvement studies, as well as production.

## Introduction

Single nucleotide polymorphisms (SNPs), the most common mutation in mammalian genomes, are single-base exchanges that naturally occur in the genome and have long been studied as valuable genetic markers, as they can affect and/or aid the selection of phenotypic traits [1,2]. Current genomic selection of production animals heavily relies on identification and genotyping of molecular markers such as SNPs [3]. Mutation mechanisms result in transitions when there are purine-purine or pyrimidine-pyrimidine exchanges and transversions in the case of purine-pyrimidine or pyrimidine-purine exchanges. SNPs have gained ground over microsatellites as useful genetic markers for genomic selection due to the need for high density markers for multifactorial traits [4].

In humans, approximately 70% of the genome is translated into RNA, but 98.5% are non-coding RNAs (ncRNA), i.e. not translated into protein [5]. Among ncRNAs, microRNAs (miRNA) of approximately 22 nucleotides act as post-transcriptional regulators of gene expression, binding primarily to the 3’UTR region of target genes by antisense complementarity in a conformation known as RISC (RNA-induced silence complex). The seed region comprises nucleotides 2–8 of the miRNA and the recognition of the target genes depends mainly on their pairing [6,7]. Following complex formation, there are two ways of translational control: target miRNA cleavage or translational inhibition [8].

In humans, the occurrence of SNPs at target sites of miRNAs on messenger RNAs (mRNAs) has been reported to alter mRNA:miRNA binding, creating or destroying targets, and potentially regulating a wide variety of diseases, including cancer (reviewed by Moszyńska [9]). Notwithstanding, the presence of SNPs can also affect the miRNA sequence itself [10,11]. In bovine, the effects of SNPs in specific miRNA sequences or miRNA target sites were reported as being related to mastitis [12] and fertility [13].

Through the use of bioinformatic tools and the largest bovine SNP database currently available (1000 Bulls Genome Project), we sought to identify SNPs that occur in miRNA sequences and to perform *in silico* predictions on the effects of these SNPs on target binding, primary miRNA formation, occurrence of miRNAs with altered target prediction in QTL regions, and the evolutionary conservation for each SNP locus.

## Materials and methods

### Bovine assembly and database

In this study, we used the *Bos taurus* UMD 3.1.1 [14] as the reference genome. SNP positioning of approximately ~56 million SNPs were acquired from the Run5 of the 1000 Bulls Genome Project (1000BGP; http://www.1000bullgenomes.com/) INDELS were not used in this study. Primary and mature miRNA location, positioning, and sequences were obtained from miRbase (www.mirbase.org), release 21 [15–21]. Positions of QTLs were extracted from the Animal QTLdb [22]. Data mining for each mature miRNA that alter target gene prediction was performed in FAANGmine (http://128.206.116.18:8080/faangmine/begin.do) [23].

### Mapping of SNPs in miRNA sequences

An in-house Python script (2.7 version) was used to identify all SNPs (Run5 of 1000BGP) that are located within all bovine miRNA sequences available on miRBase (release 21).

### Prediction of miRNA targets

The miRmap [24] package was adapted to input both miRNA reference and SNP altered sequences for target prediction through Python scripts (3.6.4 version), and using 13,345 3`UTR bovine target sequences available within the same package. Target prediction was performed for all miRNAs containing SNPs. To validate microRNA targets predicted by the miRmap software, we also used the algorithm employed by the TargetScan software [25] to perform target prediction. We used the 3’ UTR database available on the miRmap package against our miRNA reference sequences and sequences altered by SNP. Common targets, predicted by softwares, are described in Fig 2 and in supplementary S1 File. Reference sequences (similar to UMD 3.1.1) and alternate sequences (alternate allele from Run5) were then classified in regards to target gene prediction as: 1) targets were created as a result of SNP; 2) targets were lost; and 3) targets were unaltered. Welch Two Sample t-test was employed to compare transitions and transversions in regards to target alteration.

### Prediction of energy change in primary miRNA sequences

To calculate variations in Minimum Free Energy (MFE) between reference and altered sequences of primary miRNAs, the program RNAFold—ViennaRNA Package 2.0 [26] was utilized. This tool can calculate changes in MFE, which can affect the formation of mature miRNAs, based on alterations to the hairpin structure of primary miRNAs caused by unpairing of bases within the double strand section of the hairpin. Welch Two Sample t-test was used to compare transitions and transversions in regards to the MFE change induced. The threshold 2.0 applied was previously reported by Gong et al. as sufficient to alter the MFE [27].

### QTLs

An in-house Python script (version 2.7) was used to identify which SNPs, with altered target prediction, were located in QTL mapped regions available in Animal QTLdb [22].

### PhyloP scores

PhyloP scores are used to measure non-neutral nucleotide substitution rates, indicating conservation and acceleration independently at each nucleotide [28]. To calculate PhyloP scores [29], we performed a BLAST [30] search (e-value ≤ 10^−3^) [31] of all primary miRNA sequences against the human genome, then the identified human sequences were located on the UCSC Genome Browser [32,33]. Once sequences were located, an alignment was performed using MUSCLE [34], followed by manual search of the corresponding human base pair to the original bovine SNP position. Lastly, the conservation track Cons 100 Verts (phyloP100way), available on the UCSC genome browser, was used to obtain the PhyloP value to each position of interest.

## Results

### SNPs in miRNA primary sequences

**Fig 1:**
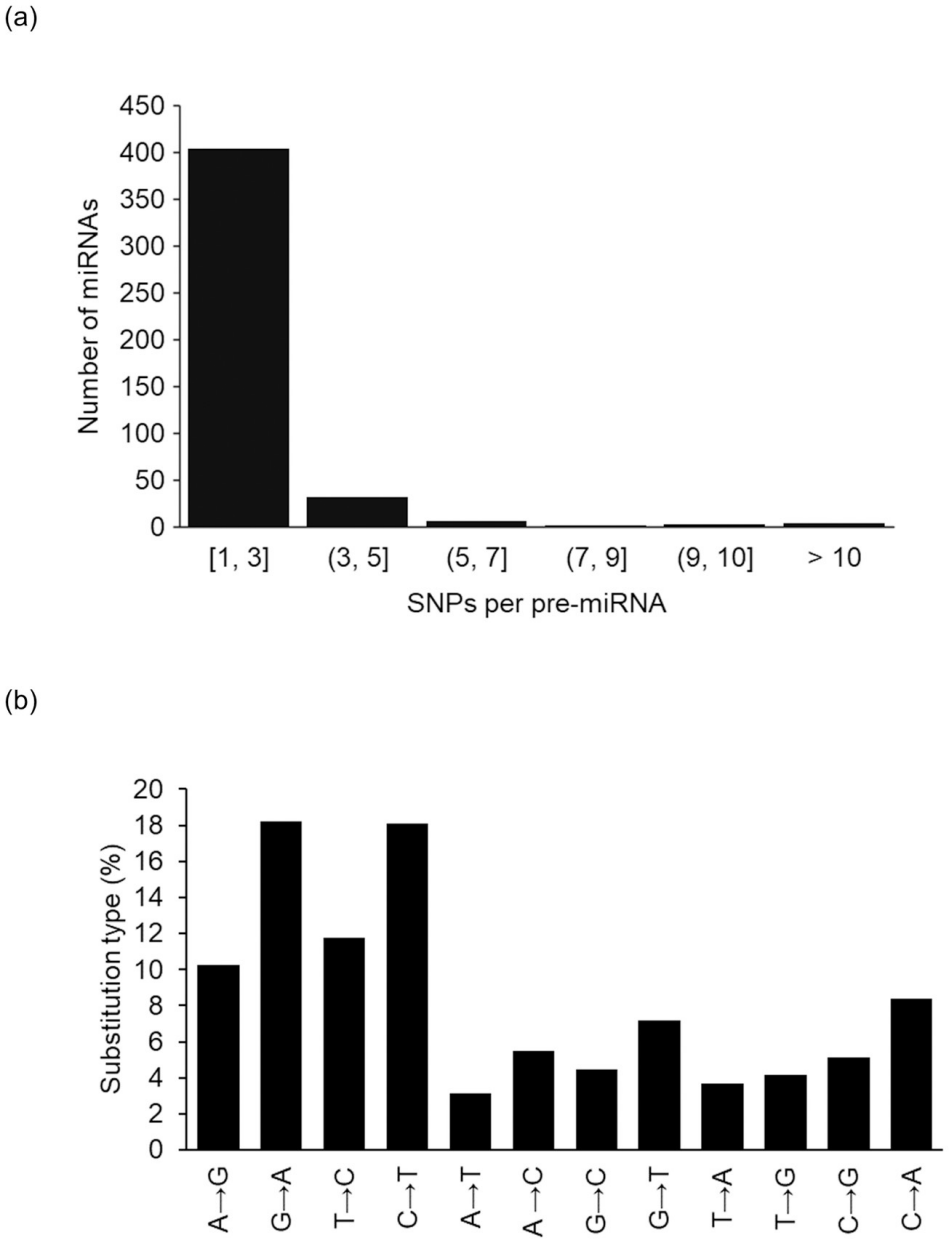
Frequency distributions of SNPs per miRNA primary sequences (a) and nucleotide substitution type (b) in primary sequences. Arrows indicate the direction of change, based on the reference genome.

We performed a scan in 1,064 miRNA primary sequences and found 452 miRNA sequences with SNPs. Out of 452 miRNAs with SNPs, 193 of those had SNP(s) located within miRNA´s mature sequences, with 91 of them within seed regions. Some miRNAs harbored more than one SNP per sequence. Nucleotide changes were comprised of 47% transitions and 53% transversions (Fig 1).

### Changes in target prediction

**Fig 2:**
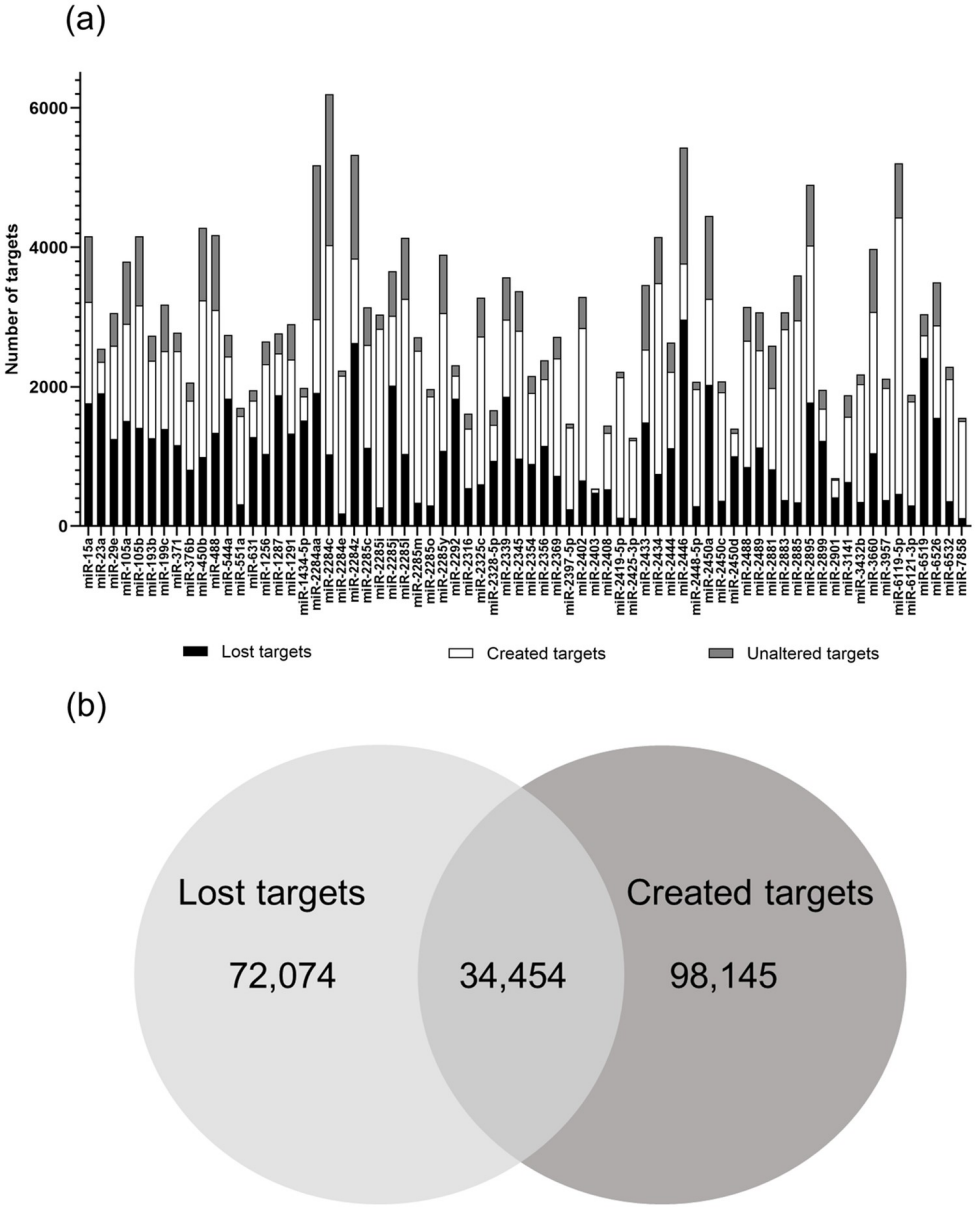
Changes in target prediction. Prediction of gained, lost and unaltered targets of mature miRNAs containing SNP (a). Venn diagram depicting all created, lost and maintained targets (b).

Out of 193 miRNA mature sequences with SNPs, 91 had SNPs occurring within their seed region, and 70 of these 91 (77%) had changes in target prediction as a consequence of the nucleotide present. In regards to target prediction alteration observed for the 70 miRNAs, 48% of all targets were newly created, and 35.1% were lost (Fig 2a and 2b), as a consequence of the presence of a SNP. Less than one third of the targets were unaltered by the presence of SNP within the seed region of the miRNAs (Fig 2b). No significant difference in target alteration was observed between transitions and transversions (1,101 vs 1,238 average target alteration, respectively). The list of target genes for reference and altered miRNAs is in S1 File.

### Effects of SNPs on miRNA hairpin structure

**Fig 3:**
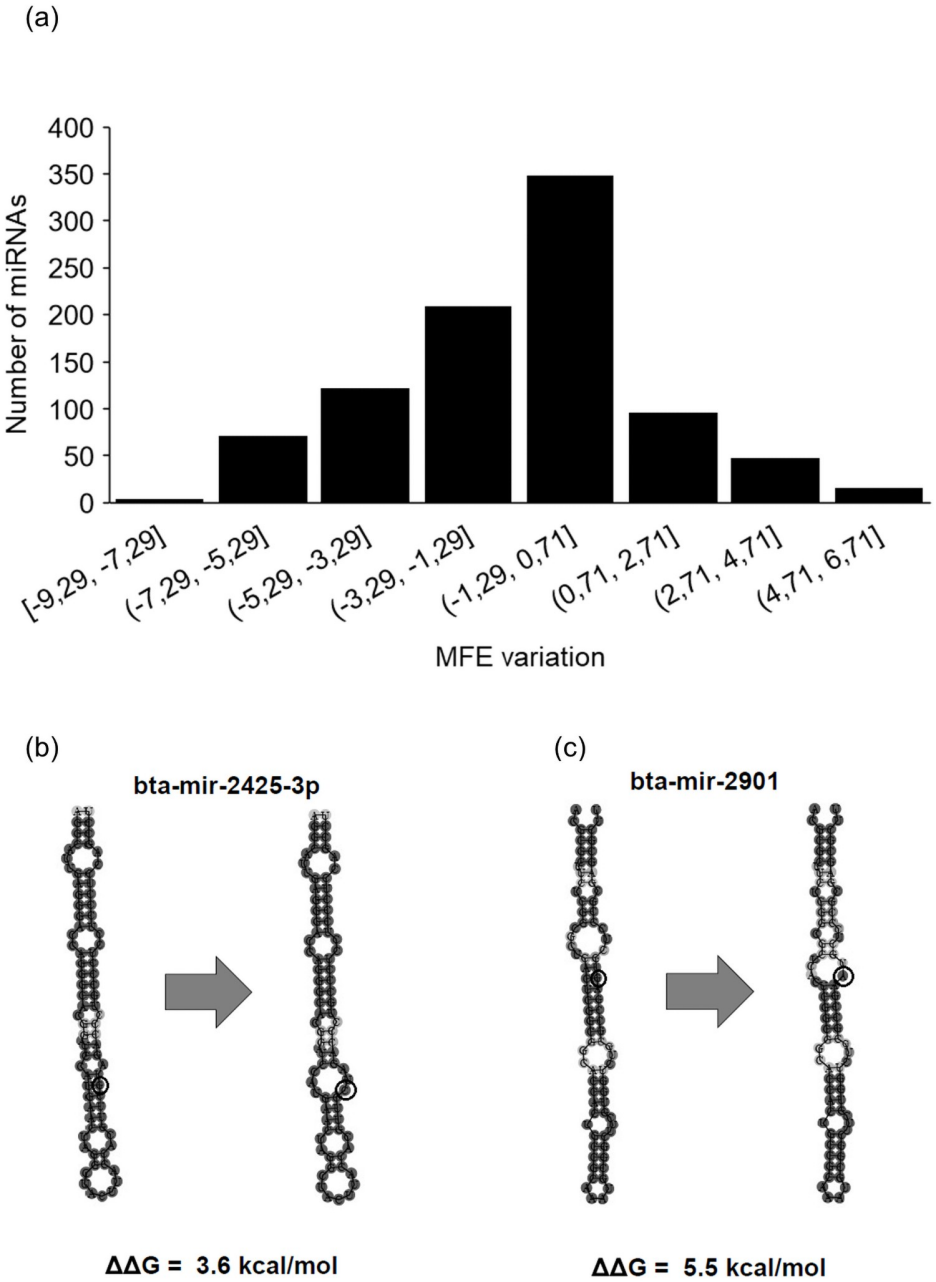
Effects of SNPs in miRNA hairpin structure. Frequency distribution of energy variation between reference and altered miRNA sequences (a). Illustration of bta-mir-2425-3p with a G to C transversion (b) and bta-mir-2901 (c).

In order to investigate the influence of a SNP in the hairpin structure of the miRNAs, we calculated the energy change (ΔΔG) between reference and altered sequences of 917 miRNA sequences (Fig 3A) using the RNAfold Program [26]. We observed that the presence of SNPs can alter hairpin conformation due to loss or acquisition of base pairing, consequently altering free energy (Fig 3B and 3C) in 434 primary miRNAs. In the present study, we found 58% transitions and 42% transversions, and statistical analysis indicated that there was a significant difference regarding MFE variation between these two types (-0.83 vs -1.61, transitions and transversions, respectively; Welch Two Sample t-test p-value<2.348e-05).

### Presence of miRNAs with SNPs in QTL regions

Focusing on the 70 mature miRNAs with altered target prediction, we crossed their positions with that of known QTL regions available on Animal QTLdb (Table 1). We found 46 traits that had one or more SNPs in 47 different miRNA sequences within their locations.

**Table 1:** Mature miRNA with SNPs affecting target prediction in QTL regions.

Trait categories	miRNA
**Reproduction**	
Calving ease	bta-miR-181c bta-miR-23a bta-miR-2881 bta-miR-2284o bta-let-7e bta-miR-574 bta-miR-2900 bta-miR-199c bta-miR-2339 bta-miR-584 bta-miR-2886
Calving ease (maternal)	bta-miR-2284i bta-miR-2285o bta-miR-551a bta-miR-2325c bta-miR-2403
Calf size	bta-miR-2414 bta-miR-2284b bta-miR-3660 bta-miR-2387
Birth index	bta-miR-2328-5p bta-miR-2901 bta-miR-2899
Stillbirth	bta-miR-199a-5p bta-miR-371
Scrotal circumference	bta-miR-2418 bta-miR-2898
Udder swelling score	bta-miR-2293 bta-miR-2892
Calving to conception interval	bta-miR-2419-5p
Age at puberty	bta-miR-6121-3p
Calving index	bta-miR-2480
Length of productive life	bta-miR-2292
**Milk production**	
Milk caproic acid content	bta-miR-2284r bta-miR-1388-5p bta-miR-1 bta-miR-2307 bta-miR-544b bta-miR-7858 bta-miR-449b bta-miR-877 bta-miR-6531 bta-miR-2284c bta-miR-219
Milk palmitoleic acid content	bta-miR-2286 bta-miR-2284y bta-miR-2354 bta-miR-488 bta-miR-669 bta-miR-6532
Milk butyric acid content	bta-miR-2284u bta-miR-181b bta-miR-2344 bta-miR-2345
Milk decenoic acid content	bta-miR-2318 bta-miR-584 bta-miR-2369
Milk myristic acid content	bta-miR-2285y bta-miR-2285o bta-miR-2400
Milk capric acid content	bta-miR-2895 bta-miR-1287 bta-miR-2285k
305-day milk yield	bta-miR-1839 bta-miR-2309 bta-miR-193a
Milk oleic acid content	bta-miR-2285l bta-miR-2356
Milk myristoleic acid content	bta-miR-1256 bta-miR-1721
Milk alpha-lactalbumin percentage	bta-miR-155 bta-miR-885
Milk lauroleic acid content	bta-miR-3432b bta-miR-1434-5p
Milk yield	bta-miR-2285o bta-miR-584
Milk beta-casein percentage	bta-miR-126-5p
Milk conjugated linoleic acid content	bta-miR-2465
**Conformational**	
Body weight (weaning)	bta-miR-2285b bta-miR-2285t bta-miR-1584-3p bta-miR-8550 bta-miR-2285u bta-miR-6519 bta-miR-2324 bta-miR-6530 bta-miR-128
Body weight (yearling)	bta-miR-302b bta-miR-2444
Residual feed intake	bta-miR-2284l bta-miR-30d
Somatic cell score	bta-miR-3141
Average daily gain	bta-miR-2466-3p
Body weight (birth)	bta-miR-2362
Conformation score	bta-miR-2402
Non-return rate (EBV)	bta-miR-2421
**Meat characteristics**	
Shear force	bta-miR-2284ab bta-miR-2446 bta-miR-455-5p bta-miR-6536 bta-miR-631 bta-miR-940 bta-miR-2398
Intramuscular fat	bta-miR-2425-3p bta-miR-1291 bta-miR-2429 bta-miR-2430 bta-miR-2433 bta-miR-2434
Tridecylic acid content	bta-miR-2313-3p bta-miR-6518 bta-miR-2284d bta-miR-2316 bta-miR-144
Margaric acid content	bta-miR-2885 bta-miR-2406 bta-miR-2408
Subcutaneous fat	bta-miR-2489
Muscle iron content	bta-miR-6533
Trans-15-C18:1 fatty acid content	bta-miR-2305
Monounsaturated fatty acid content	bta-miR-653
Cis-10 Heptadecenoic acid content	bta-miR-2381
**Environmental adaptation**	
Cold tolerance	bta-miR-6120-3p bta-miR-2284y bta-miR-7691 bta-miR-2325a
Heat tolerance	bta-miR-2397-5p
**Disease tolerance**	
Tick resistance	bta-miR-2284z bta-miR-199a-5p
Bovine tuberculosis susceptibility	bta-miR-2883

### PhyloP conservation scores for SNPs within primary sequences

**Fig 4:**
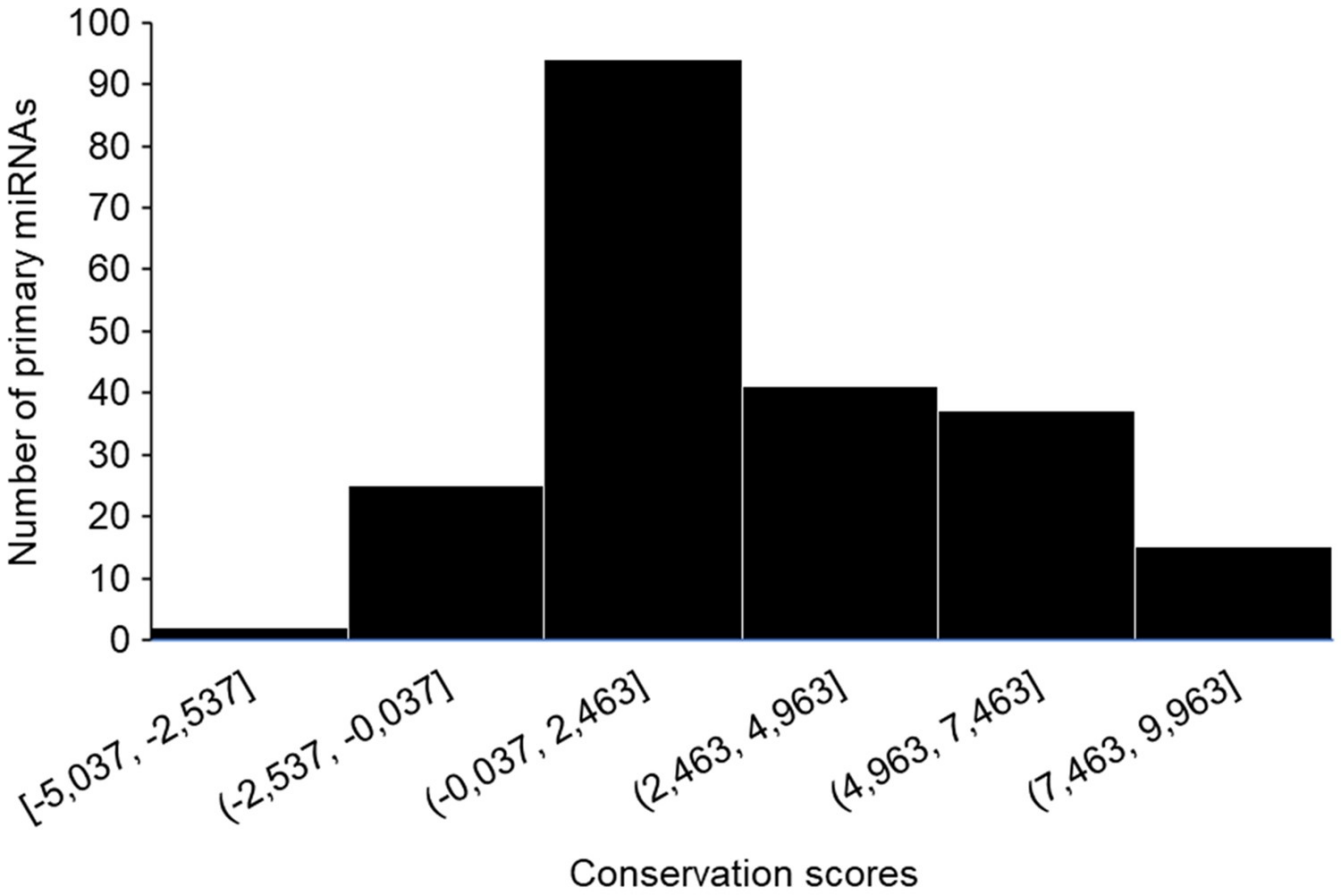
PhyloP conservation scores. Frequency distribuition of PhyloP scores for each SNP position in miRNA sequences. Positive, neutral and negative values are employed to indicate slow, neutral and fast-evolving variations, respectively.

To investigate the conservation level of each altered base occurring in miRNA primary sequences, we obtained the PhyloP score based on the alignment of 100 vertebrates [29] relative to human genome. Positive, neutral and negative values are employed to indicate slow, neutral and fast-evolving variations, respectively. Of the 917 primary miRNAs sequences containing SNPs, we were able to confidently align 214 bovine sequences to homologous sequences in humans, in order to obtain the phyloP score of each SNP position (Fig 4). All miRNAs containing a SNP in their seed region had a positive phyloP score, regardless of target alteration.

## Discussion

We were able to contrast the largest SNP database available in the bovine species to date, with all available bovine miRNA sequences. The publicly available SNP databases, Bovine Genome Database [35] and SNPchiMp v.3 [36,37], that gathers data from SNPs used on several chips produced by Illumina and Affymetrix, have a combined total of 2 million SNP annotations, whereas the dataset used in this study, Run 5 of the 1000BGP, has more than 56 million SNP annotations.

In our study, SNPs within seed sequences greatly affected target prediction, with less than 20% of similar targets between miRNAs containing the reference or altered alleles [38]. Our prediction demonstrated a large number of targets for each miRNA, as observed in humans, and given that 90% of human genes are estimated to be regulated by miRNAs [39], we suggest that SNPs within miRNAs may have a significant impact on cattle phenotypes. Target gain and loss due to the presence of SNPs in microRNA sequences or miRNA binding sites have been previously reported for 41 human miRNAs, affecting approximately 50% of their predicted targets [40]. Luciferase assay confirmed loss of target-binding in 7 of these miRNAs, as well as target gain in 1 of the evaluated miRNAs [40]. In another study, 30 million SNPs were mapped to 1048 human pre-miRNAs, and target prediction detected a wide variation between reference and altered miRNAs, with an average gain of 1,790 targets among the three miRNAs with the highest gains, and of 1,576 targets lost among the three with the highest losses [27]. This previously study also validated 4 miRNAs that lost targets and 1 miRNA that gained targets, by luciferase assays, confirming the prediction. Our results in cattle suggest that the same biological mechanisms are applicable to both species as we demonstrate a major impact on target prediction due to the occurrence of SNPs in the miRNAs seed region. Changes in prediction of miRNA targets as a consequence of SNPs are not limited to the animal kingdom. In plants, a 26% target gain was observed for 13 miRNAs with SNPs in their seed region [41].

We found a significant increase in the variation of MFE in the transversions when compared to transitions. This variation was also reported by Guo et al. [42], however, their study considered only SNPs in coding regions, so the present study suggests that the type of substitution also interferes with the regulatory effects of non-coding RNAs. Presence of SNPs in primary miRNAs sequences, such as observed in 434 miRNAs in our study, may also alter the production of mature miRNAs. This is due to alterations in recognition by their cleavage proteins, which identify the conformation of the secondary structure of the molecule and not simply the nucleotide sequence [43,44]. In fact, association between the presence of SNPs in the pre-miRNA flanking region and cancer has been reported in humans [45]. Alteration in MFE in miRNA primary sequences, caused by SNPs, are also reported and validated in carp (*Cyprinus carpio*) [46]. Positive values greater than 2.0 account for reducing mature miRNA production and negative values for increasing mature miRNA production in humans and viruses (reviewed by Gong et al. 2012) [27]. In our study 48.6% of miRNAs primary sequences with SNPs showed ΔΔG greater than this value. MFE alteration between reference and altered pre-miRNA-2467 has already been reported in a study with five bovine breeds (Angus, Jersey, Holstein-Friesian, Limousin, Hereford), where a loss of stability resulted from the formation of a new loop in the hairpin structure following a loss of base pairing [47].

In our study, we have identified 125 miRNAs with SNPs in previously identified QTL regions. These loci are large, spanning several Kbs within chromosomes, therefore caution should be taken when discussing point variations in such large regions. Notwithstanding, 47 of these miRNAs associated with QTLs presented altered target prediction as a consequence of a SNP within their seed region, which could translate directly in significant protein differences in individuals harboring different bases in non-coding regions of the genome. The influence of SNPs on the divergence of traits between cattle breeds, or within breed, are well reported in several studies, as revised by Seidel [48], however non-protein-coding sequences, outside of promoter regions, have been largely overlooked as possible functional variations in cattle. One of the few studies associating such regions to phenotypes is a published GWAS analysis that detected the presence of two SNPs within the same LD block (separated by 80 kb) that, together, were related to calving ease. This region is devoid of protein coding genes, but contains a miRNA, bta-mir-1256 [49]. Interestingly, our prediction of target gene binding indicated that a SNP present in bta-miR-1256 causes this miRNA to target *LCORL* for downregulation. The *LCORL* gene encodes the PALI1 and PALI2 proteins, essential for the activation of the PRC2.1 and PRC2.2 complexes and regulation of the suppressive histone mark H3K27me3, essential for the normal development, as demonstrated in mice [50]. The *LCORL* gene is highly conserved among mammals and is related to skeletal height and size in humans [51–53], body size in horses [54–57], dogs [58] and pigs [59], body weight in chickens [60,61] and sheep [62]. Previous studies in cattle have linked the *LCORL* gene with body conformation indexes in Nordic red cattle [63], carcass weight [64] and organ weight [65] in Simmental cattle, growth in Quinchuan cattle [66] and stature in Limousin, Hereford and Angus [67], thus, suggesting that the acquisition of regulation of *LCORL*, caused by a SNP in bta-miR-1256, may affect overall size and therefore calving ease.

In addition, Purfield et al. [31] detected a SNP close to the *IGFBP-2* gene in a QTL related to calving difficulty. Human growth hormone (GH) regulates the expression of IGF (insulin-like growth factor) and the deficiency of GH / IGF1 results in short stature in children. IGFBP (Insulin-like growth factor-binding protein) belong to a group of 6 proteins that bind to IGF and have as main function to regulate the access of IGF1 and 2 to the IGF1 receptor [68,69].

Evolutionary conservation of miRNAs, as well the conservation of their target genes, have already been evidenced in previous studies [70,71]. In this study we decided not to limit ourselves to determining the conservation of miRNA sequences based on homologous occurrence in other vertebrates, but rather to infer conservation relevance by retrieving phyloP scores. PhyloP scores with positive values represent conservation, while scores with negative values represent rapid evolution. Our analysis showed that ~86% of the loci of the 214 aligned miRNA sequences, where SNPs occurred, had scores greater than 0, suggesting a conserved biological function through the evolutionary process, where natural selection favored individuals who maintained this conserved position, increasing their frequency in populations. Therefore, a change in these conserved positions could suggest unfavorable phenotypes, influenced by altered maturation of primary miRNAs or misregulated post-transcriptional repression promoted by mature miRNAs.

Among the miRNAs with altered target-binding prediction, we highlight miR-15a and 23a, which have their binding to target genes previously validated in cattle. MiR-15a targets the *CD163* gene and was recently identified by Ju *et al*., where increased expression levels of these miRNAs in the udder of Holsteins were associated with mastitis [72]. Interestingly, our study revealed that a SNP present in the seed region in miR-15a is predicted to alter its binding with *CD163*, which could influence immune response to infection. In 2017, Guan et al. identified the *ZNF423* gene as a target for miR-23a, affecting cellular differentiation in bovine fetal muscle. Authors reported that the level of expression of miR-23a is low during cell differentiation, while the forced overexpression of a miR-mimic 23a leads to a reduction in the accumulation of lipids [73]. Gupta el at. (2010) showed that *ZNF423* acts as a positive regulator of PPARγ expression, promoting the differentiation of undifferentiated cells into adipocytes. They have also demonstrated that the reduction of *ZNF423* expression, through small RNAs, negatively regulates the rate of adipocyte differentiation [74]. In the present study, a SNP in the miR-23a resulted in the loss of *ZNF423* as a target, and in the gain of *ZNF521* which, interestingly, was previously reported as a negative regulator of *ZNF423* in mice [75]. Thus, the loss of *ZNF423* as a target for miR-23a, in addition to downregulation of *ZNF521*, caused by the presence of a SNP in the miR-23a seed region, can result in increased adipogenesis and could impact meat marbling.

A phenome search carried out through the FAANG (Functional Annotation of Animal Genomes) Consortium [23], using our 70 miRNAs with altered target prediction, pointed to a role for miR-29e on immune response. Lawless et al. [76] predicted 2,491 target genes for differentially expressed miRNAs identified after infection by *Streptococcus uberis* in bovine mammary epithelial cells. A pathway analysis highlighted MAPK signalling, Cytokine-Cytokine Receptor Signalling and the JAK-STAT Signalling Pathway as important pathways for mastitis response in these cells. Of the 2,491 target genes predicted by the authors, 93 genes were predicted as targets for mir-29e, which was upregulated at 4 hours of infection and downregulated at 6 hours. When we compare these targets with the prediction performed in our study, the presence of a SNP in the miR-29e seed region results in the loss of 52 of these 93 target genes and a gain of 4 target genes, suggesting that a SNP in the seed region of this miR-29e miRNA can alter more than 50% of the target genes for this miRNA, as predicted by Lawless et al., potentially affecting important pathways related to innate immunity in response to gram-positive bacterial infection.

Finally, the present study points to an approach focused on differences in genomic sequences that are not present in coding genes, but can indirectly generate differences in the phenotype of individuals, through epigenetically regulated processes. In fact, our group has previously identified SNPs that, through the creation or destruction of CpG sites, may mechanistically induce a quantitative difference in methylation, which in turn modulates gene expression and consequently phenotypes [77]. Out of 1,064 bovine miRNAs presently described in miRbase, only 265 have target genes that have been experimentally validated [78] and, of these, only 3.7% have targets within their own chromosome. This further highlights the importance of considering point changes away from protein-coding regions, as those can affect genes located in distant loci and indirectly explain phenotypic variation between animals through epigenetic regulation.

## Conclusions

In the present study we demonstrated that SNPs in miRNA sequences can alter their target prediction, as well as pre-miRNA processing, possibly increasing or decreasing mature miRNA production, which in turn could affect production traits in cattle.

## Supporting information

S1 File List of target genes predicted for reference and altered miRNAs. (XLSX)
